# Atypical Presentation of Acquired Hypoganglionosis in a Patient Taking Clozapine

**DOI:** 10.1155/2020/1272567

**Published:** 2020-06-24

**Authors:** Catherine Gisi, Anamaria Munteanu, Lawrence Ku, Samuel French, Michael Fleischman, Viktor Eysselein

**Affiliations:** ^1^Department of Internal Medicine, Harbor-UCLA Medical Center, Torrance, CA, USA; ^2^Department of Pathology, Harbor-UCLA Medical Center, Torrance, CA, USA; ^3^Department of Internal Medicine, Division of Gastroenterology, Harbor-UCLA Medical Center, Torrance, CA, USA

## Abstract

Acquired hypoganglionosis (HG) is a rare enteric gastrointestinal neuromuscular disorder previously associated with chronic inflammation that can lead to constipation, ileus, and even death. There is little known about the pathophysiology of acquired hypoganglionosis, and it is unclear if medications are related to the development of the disease. Clozapine is an atypical antipsychotic used to treat refractory schizophrenia that is well known for its side effects including agranulocytosis and gastrointestinal dysmotility. This is an unusual case of acquired hypoganglionosis in a patient with anticholinergic toxicity on clozapine therapy.

## 1. Introduction

Normal gastrointestinal motility depends on appropriate enteric nervous system signaling and coordinated smooth muscle contraction [1]. The enteric nervous system is the largest and most intricate component of the peripheral nervous system. The myenteric (Auerbach's) and submucosal (Meissner's) plexuses contain the majority of neurons and ganglia in the gastrointestinal tract [2]. Acquired hypoganglionosis (HG) is a rare gastrointestinal neuromuscular disease characterized by a decreased number of ganglia in the myenteric and submucosal plexuses [3, 4]. In contrast to congenital hypoganglionosis, such as in Hirschsprung's disease, the acquired form presents in adolescence or in adulthood. Histopathology characteristically demonstrates thickened muscularis mucosa and muscularis propria with decreased numbers of myenteric ganglia [3]. There are multiple theories regarding the pathophysiology of HG. It is primarily thought to be caused by chronic inflammation in the colon [3]. No medications have yet been associated with development of the disease. Differential diagnosis of acquired hypoganglionosis includes chronic intestinal pseudo-obstruction, rare enteric myopathies (mutations of the ACTG2 gene), Hirschprung's disease, Chagas disease, enteric neuropathies, and Waardenburg–Shah syndrome [5, 6].

Clozapine is an atypical antipsychotic that is regarded as the most effective treatment for refractory schizophrenia. Despite its efficacy, clozapine remains a third-line medication due to its side effects, including agranulocytosis and severe constipation leading to ileus, gastrointestinal necrosis, and perforation [7]. In January 2020, the FDA strengthened its warning that clozapine-related ileus can lead to serious complications. A review by Cohen et al. reported the case-fatality rate for clozapine-related lower gastrointestinal hypomotility to be 15–27.5%, which is about 7 times that of clozapine-related agranulocytosis [8]. Another review reports a mortality rate of 43.7% due to clozapine-induced ileus from cumulative case reports [9]. Though the mechanism is unknown, it is postulated that clozapine causes constipation due to its anticholinergic, serotonergic, and antihistaminergic properties, similar to other antipsychotics [10].

Presented is a case of acquired hypoganglionosis found in a patient with severe ileus in the setting of the anticholinergic toxicity attributed to clozapine.

## 2. Case Report/Case Presentation

A 45-year-old Caucasian male with a history of schizoaffective disorder and chronic constipation was brought to the emergency department for acute encephalopathy. He was somnolent and unresponsive to questions. His home medications included a total of 1,200 mg of clozapine daily. It is unclear how long he was taking this dose. He was also taking risperidone, propranolol, linaclotide, benztropine, alprazolam, and diphenhydramine. The patient's medical team could not determine exactly how many years the patient had been taking clozapine, but family and the patient's medical team indicated that he was started on the clozapine therapy years ago in his adulthood. He had never been diagnosed with ileus or other complications of constipation. He had no surgical history. On arrival, the patient was febrile to 38.5°Celsius and tachycardic but otherwise hemodynamically stable. Initial abdominal examination was soft, nontender, mildly distended with reduced bowel sounds.

Laboratory studies showed a clozapine level of 735 mcg/L and norclozapine level of 449 mcg/L (reference value normal 25–440 mcg/L). Other labs were unremarkable except for a hemoglobin level of 11.8 g/dL. Cerebrospinal fluid, blood cultures, and urine cultures were negative. Computed tomography (CT) of the head without contrast was unremarkable. He was found to have significant urinary retention on bladder ultrasound. Given the negative infectious workup and his clinical presentation, along with his multitude of medications with anticholinergic side effects, a working diagnosis of anticholinergic toxicity was made, and the patient's home clozapine was held.

On hospital day 2, he developed bilious emesis and physical exam revealed a soft, distended, and tympanic abdomen that was mildly tender. Abdominal CT showed a large stool burden diffusely in the small and large intestines without obvious transition points that was concerning for ileus. The patient underwent aggressive medical therapy including frequent tap water enemas and polyethylene glycol administered via nasogastric tube and per rectum. He remained NPO (nulla per os) and underwent intermittent nasogastric tube suctioning. Large amounts of stool were evacuated, but the patient continued to have bilious vomiting. Repeat abdominal CT showed fecalization of stool in the small intestine and dilatation of the proximal and rectosigmoid colon to 8.4 cm and 7 cm, respectively, without a clear transition point. The patient was transferred to the intensive care unit and treated with two doses of intravenous neostigmine with significant evacuation of stool. Repeat abdominal CT demonstrated bowel without stool but with significantly worsened dilatation. The patient underwent total colectomy with end ileostomy. Given the remarkable dilatation of the rectum, there was concern that primary ileorectal anastomosis would have led to an inadequate functional outcome, and the decision was made to perform an end ileostomy. The patient recovered rapidly to his mental and functional baseline postoperatively with resumption of normal bowel movements.

Given the patient's severe psychiatric symptoms refractory to other medications, a multidisciplinary meeting with psychiatry was held and it was decided to slowly restart the patient on clozapine with monitoring of symptoms and drug levels. Clozapine was titrated slowly with close monitoring for side effects. The repeat serum clozapine and norclozapine levels were 433 mcg/L and 121 mcg/L, respectively. There was no recurrence of systemic clozapine toxicity or symptoms of gastrointestinal dysmotility at these lower levels.

**Figure 1:**
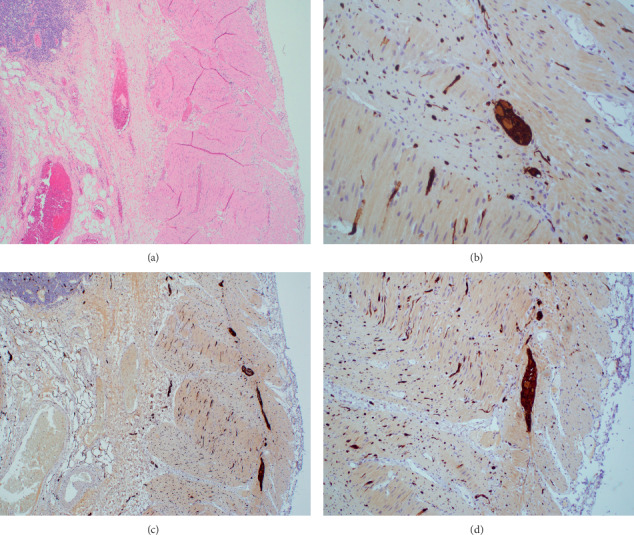
Ileum. (a) Hypertrophy of muscularis propria (magnification 10x, H&E). (b) Focally absent ganglion cells (magnification 20x, S100). (c) Auerbach's plexus highlighted in dark brown (magnification 10x, S-100). (d) Numerous ganglion cells in some areas (magnification 20x, S100).

**Figure 2:**
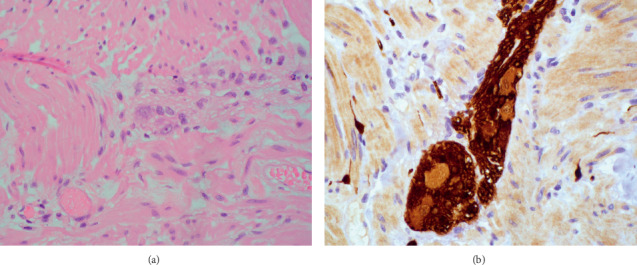
Cecum. (a) Reduced ganglion cells (magnification 40x, H&E). (b) Reduced ganglion cells (magnification 40x, S100).

**Figure 3:**
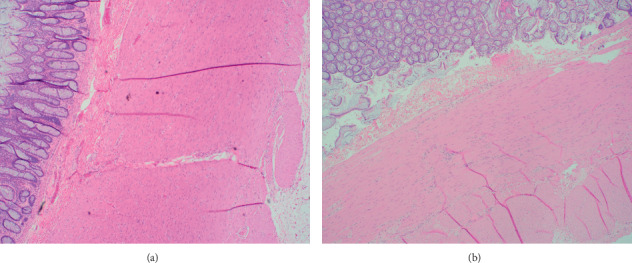
Hypertrophy of the muscularis propria in rectum and sigmoid sections. (a) Rectum (magnification 10x, H&E). (b) Sigmoid colon (magnification 10x, H&E).

**Figure 4:**
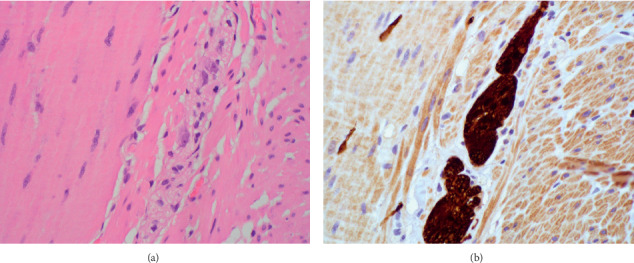
Sigmoid colon. (a) Focally absent ganglion cells (magnification 40x, H&E). (b) Focally absent ganglion cells (magnification 40x, S100).

Full-thickness sections from the ileum (Figure 1), cecum (Figure 2), appendix, colon, sigmoid, and rectum (Figures 3 and 4) were obtained and stained with hematoxylin and eosin (H&E), calretinin, and for S-100, a neuronal marker. Final pathologic diagnosis showed hypertrophy of the colonic muscularis propria in the rectum, sigmoid, and cecum. There was focal loss of muscularis propria in the proximal colon. Though there were some ganglia present in the myenteric and submucosal plexuses, they were only focally present in the proximal colon and appendix. Sections from the ileum showed similar muscle hypertrophy and focally absent ganglia in both plexuses consistent with acquired HG.

## 3. Pathology

Full-thickness sections from the ileum, cecum, appendix, colon, and rectum were obtained and stained with hematoxylin and eosin (H&E) and for S-100, a neuronal marker.

Final pathologic diagnosis showed hypertrophy of the colonic muscularis propria in the rectum, sigmoid, and cecum. There was focal loss of muscularis propria in the proximal colon. Though there were some ganglia present in the myenteric and submucosal plexuses, they were only focally present in the proximal colon and appendix. Sections from the ileum showed similar muscle hypertrophy and focally absent ganglia in both plexuses. Findings were consistent with acquired hypoganglionosis.

## 4. Discussion

There are approximately 100 reported cases of isolated HG distinct from Hirschsprung's disease in the literature in the last 40 years, and only a few of those cases were in Caucasian adults [3, 11]. It is also associated with considerable morbidity and mortality [5, 11]. The prevalence of the disease is difficult to quantify because diagnosis is made on histology, which requires examination of full-thickness bowel sections [5]. Immunohistochemistry stains for acetylcholinesterase, S-100, interstitial cells of Cajal, silver, and lactate dehydrogenase are essential in making the diagnosis [11]. It is distinctly different from congenital HG (Hirschsprung's disease), which is diagnosed in children with characteristic contiguous aganglionic segments that start at the anal sphincter and extend proximally [2].

Chronic inflammation in the colon and lower gastrointestinal tract is a common theme unifying theories behind the pathophysiology of acquired HG [3]. Infections, autoimmune diseases, and inflammatory bowel diseases have all been postulated as etiologies of chronic inflammation leading to gut mucosal breakdown and formation of autoantibodies to ganglion cells [3]. Though antipsychotics have prominent anticholinergic effects and have been known to cause constipation and diarrhea, they have not been previously linked to inflammation [9].

It has been reported that acute infection may dramatically increase the serum concentration of clozapine through downregulation of cytochrome *P*450 enzymes during inflammation and infection [12]. In this case, no obvious signs of infection were found, but similar cases of severe ileus in the setting of clozapine may warrant close investigation into possible concurrent infections. There are various data on the optimal therapeutic serum levels of clozapine and its more active metabolite, norclozapine [13, 14]. Serum levels between 350 and 1000 mcg/L have been associated with greater efficacy and less toxicity, though patients may experience toxicity at various levels, and titration should be individualized with careful monitoring of side effects and symptoms [14]. In this patient, initial higher levels of clozapine and its metabolite, norclozapine, were associated with severe side effects, which were no longer present when clozapine was stopped. With careful titration and monitoring, the patient achieved lower serum concentrations of clozapine without suffering severe side effects, though this is difficult to interpret because he was postcolectomy.

The Naranjo scale is a validated scoring system for estimating the probability of an adverse drug reaction. In this case, the Naranjo score is 7, indicating a probable adverse drug reaction [15].

Very few cases of acquired HG have been reported, but the typical presentation of the disease is severe adult-onset ileus with significant colonic distension resulting in colonic necrosis, perforation, and curative colectomy. Pathology in this case did not show classic signs of chronic inflammation but was consistent with the typical clinical and histopathologic presentation of acquired hypoganglionosis. It also overlaps with the well-documented presentation of clozapine-related ileus. Despite a large body of literature on clozapine-related ileus, there is a scarcity of data describing the histopathology of this disease [16].

There are numerous reports of fatal clozapine-related ileus in the literature [17]. It is thought that clozapine significantly reduces gastrointestinal motility primarily by affecting the peripheral muscarinic anticholinergic activity via the M3 receptors in the gut wall [7]. Clozapine is three times more likely to cause constipation than other antipsychotics [5]. One recent study demonstrated that 82% of patients taking clozapine had dysmotility in at least one region of the GI tract, with more than half of those patients experiencing multiregional dysmotility [18].

In this case, the patient had suffered from constipation for most of his adult life. He had no known history of severe constipation as an adolescent, making congenital aganglionosis unlikely. Given the prevalence of clozapine-induced dysmotility and his severe manifestations of constipation later in adulthood, it is likely that his constipation was caused by years of clozapine and antipsychotic use. The mechanism of clozapine-induced constipation is unknown and difficult to study. One challenge is obtaining tissue for analysis from patients who suffer from clozapine-related dysmotility and ileus. Colon biopsies are relatively invasive and difficult to obtain. It is possible that many patients taking clozapine have acquired hypoganglionosis but remain undiagnosed because tissue analysis and special stains for ganglionic cells are rarely performed. In this case, this patient would have remained undiagnosed if he had not had catastrophic complications and a subsequent colectomy. More research is needed to determine the pathophysiologic mechanism behind clozapine-induced ileus and the possibility that it is related to acquired hypoganglionosis.
